# Genetic risk for hospitalization of African American patients with severe mental illness reveals HLA loci

**DOI:** 10.3389/fpsyt.2024.1140376

**Published:** 2024-02-26

**Authors:** Adriana Lori, Brad D. Pearce, Seyma Katrinli, Sierra Carter, Charles F. Gillespie, Bekh Bradley, Aliza P. Wingo, Tanja Jovanovic, Vasiliki Michopoulos, Erica Duncan, Rebecca C. Hinrichs, Alicia Smith, Kerry J. Ressler

**Affiliations:** ^1^ Department of Psychiatry and Behavioral Sciences, Emory University, Atlanta, GA, United States; ^2^ Department of Population Science, American Cancer Society, Atlanta, GA, United States; ^3^ Department of Epidemiology, Rollins School of Public Health, Atlanta, GA, United States; ^4^ Department of Gynecology and Obstetrics, Emory University, Atlanta, GA, United States; ^5^ Department of Psychology, Georgia State University, Atlanta, GA, United States; ^6^ Mental Health Service Line, Department of Veterans Affairs Health Care System, Decatur, GA, United States; ^7^ Department of Psychiatry and Behavioral Neuroscience, Wayne State University, Detroit, MI, United States; ^8^ Department of Psychiatry, Harvard Medical School and McLean Hospital, Belmont, MA, United States

**Keywords:** severe mental illness, GWAS, African American, hospitalization, HLA

## Abstract

**Background:**

Mood disorders such as major depressive and bipolar disorders, along with posttraumatic stress disorder (PTSD), schizophrenia (SCZ), and other psychotic disorders, constitute serious mental illnesses (SMI) and often lead to inpatient psychiatric care for adults. Risk factors associated with increased hospitalization rate in SMI (H-SMI) are largely unknown but likely involve a combination of genetic, environmental, and socio-behavioral factors. We performed a genome-wide association study in an African American cohort to identify possible genes associated with hospitalization due to SMI (H-SMI).

**Methods:**

Patients hospitalized for psychiatric disorders (H-SMI; n=690) were compared with demographically matched controls (n=4467). Quality control and imputation of genome-wide data were performed following the Psychiatric Genetic Consortium (PGC)-PTSD guidelines. Imputation of the Human Leukocyte Antigen (HLA) locus was performed using the HIBAG package.

**Results:**

Genome-wide association analysis revealed a genome-wide significant association at 6p22.1 locus in the ubiquitin D (*UBD/FAT10*) gene (rs362514, p=9.43x10^-9^) and around the HLA locus. Heritability of H-SMI (14.6%) was comparable to other psychiatric disorders (4% to 45%). We observed a nominally significant association with 2 HLA alleles: HLA-A*23:01 (OR=1.04, p=2.3x10^-3^) and HLA-C*06:02 (OR=1.04, p=1.5x10^-3^). Two other genes (*VSP13D* and *TSPAN9*), possibly associated with immune response, were found to be associated with H-SMI using gene-based analyses.

**Conclusion:**

We observed a strong association between H-SMI and a locus that has been consistently and strongly associated with SCZ in multiple studies (6p21.32-p22.1), possibly indicating an involvement of the immune system and the immune response in the development of severe transdiagnostic SMI.

## Introduction

Mood disorders such as major depressive disorder (MDD), bipolar disorder (BD), posttraumatic stress disorder (PTSD), along with schizophrenia (SCZ), and other psychotic disorders constitute serious mental illnesses and are the most common mental disorders leading to inpatient psychiatric care for adults. The large personal and financial burden of severe mental illness (SMI) and repeated hospitalizations calls for better strategies and techniques to understand the underlying risk factors. These disorders are heterogeneous with variable phenotypes, which make them challenging to diagnose prior to the onset of severe symptoms and difficult to treat effectively. Another commonality among these SMIs is that they each exist on a spectrum of severity, with the more acute or recalcitrant manifestations often requiring hospitalization.

Nosological characterization of major mental health syndromes, such as SCZ, BD, and MDD, arose based on their symptom patterns and course of illness. However, doubts remain about the degree of overlap of these disorders and whether they could really be categorized as entirely distinct entities. Family studies have also shown that some of these disorders, such as SCZ and BD, partially share common genetic risks, challenging their diagnostic classification (1–4). Identifying pleiotropic mechanisms, which may be responsible for these different symptom patterns, could facilitate the understanding of these psychiatric disorders.

To reach this goal, we clustered these disorders together, based on exacerbations of symptoms requiring inpatient care, to identify a common genetic etiology.

In the past years, the US has observed a growing number of hospitalizations for mental health/substance use compared to a shrinking number of hospitalizations for all other disease categories (5). Similarly, the percentage distribution of inpatient stays in the hospital increased only for psychiatric patients, while all the other categories remained stable. Hence, the need for more studies that focus on identifying the biological mechanisms causing hospitalization in psychiatric patients, facilitating the reduction of this disparity.

Due to the lack of studies in this area, the risk factors that induce SMI and hospitalization are mainly unknown but likely involve a combination of genetics, environmental factors, and socio-behavioral influences.

Recent genome-wide association studies (GWAS) have highlighted both a) the polygenic architecture of each of these psychiatric disorders (e.g., SCZ, MDD, PTSD, BD) and b) their overlapping genetic risk factors (6–9). Furthermore, cross-disorder analysis of brain structure from ENIGMA (Enhancing Neuro Imaging Genetics Through Meta Analysis) (10) identified shared morphometric signature of psychiatric illnesses, highlighting the opportunity for transdiagnostic treatments (11).

Despite the recognition of these genetic overlaps between different diseases, to our knowledge, no studies had aimed to identify genetic variants that could contribute to increasing the overall risk of developing severe psychiatric disorders, independently from their nosological characterization. Furthermore, a novel multivariate GWAS of eight psychiatric symptoms identified two transdiagnostic liabilities that distinguish between common forms of psychopathology versus rarer forms of serious mental illness, suggesting that the severity of mood and psychotic symptoms present in SMI may reflect a difference in kind rather than merely in degree (12). In this study, we selected a specific subset of patients from a trauma-exposed inner city population cohort [the Grady Trauma Project cohort (13)]. All these are of African ancestry and had mental illnesses (e.g., SCZ, BD, PTSD, MDD), with symptoms severe enough to require hospitalization (H-SMI). We then performed a GWAS to identify specific genetic loci not associated with a specific psychiatric disease but rather with a group of symptoms/dimensions shared by different conditions that lead to hospitalization (H-SMI). We then used a polygenic risk score (PRS) to assess the association with brain structures (ENIGMA) to identify possible target brain regions for severe mental illnesses.

## Methods

### Participants

The subjects for this study were part of a larger investigation of genetic and environmental factors in a predominantly African American (AA) urban population of low socioeconomic status to determine how those factors may modulate the response to stressful life events. For the current study, we selected AA participants (N=5157, 75% females) based on Ancestry principal components. Research participants were approached in the waiting rooms of primary care of a large public hospital (Grady Memorial Hospital in Atlanta, Georgia) while either waiting for their medical appointments or waiting with others who were scheduled for medical appointments. Screening interviews, including the participants’ demographic information (e.g., self-identified race, sex, and age) and psychiatric history (e.g., hospitalization, suicide attempt, and substance use behavior), were completed on-site. Further details regarding the Grady Trauma Project (GTP) dataset can be found in Gillespie et al. (13).

### Phenotype assessments

Hospitalization (“Have you ever been hospitalized for psychiatric or mental health treatment?”), SCZ treatment and suicide attempt history were assessed based on self-report (yes/no) when obtaining demographic information. Study participants completing the initial interview were invited to participate in a secondary study phase in which additional detailed measures were administered. During this secondary study phase, subjects completed additional self-report measures and structured clinical interviews, including the CAPS and SCID-DSMIV. PTSD symptom severity (PSS) was measured using the 17-item self-report scale (14). Depressive symptoms were assessed using the Beck Depression Inventory BDI (15).

Written and verbal informed consent was obtained from all participants, and all procedures in this study were approved by the institutional review boards of Emory University School of Medicine and Grady Memorial Hospital, Atlanta, Georgia.

### Genotyping

DNA was extracted from saliva collected in Oragene collection vials (DNA Genetec Inc, Ontario, Canada) using the Unadvanced kit (Beckman Coulter Genomics, Danvers, MA) or from blood using either the E.Z.N.A. Mag-Bind Blood DNA Kit (Omega Bio-Tek, Inc., Norcross, GA) or Archive Pure DNA Blood Kit (5 Prime, Inc, Gaithersburg, MD). Genotyping was performed using the Omni-Quad 1M Bead Chip (Illuminations). Genome Studio Genotype software (Illumina.Inc) was used to cluster all experiments to minimize the batch effect. Quality control was performed using the Psychiatric Genomics Consortium PTSD Workgroup guidelines (16). Briefly, Speights software17 (17) was used to assign ancestry: a small number of non-African Ancestry individuals (European and Others) were removed from our analyses. PLINK (18) was used to perform quality control analyses such as SNPs that had a call rate < 95% were removed; individuals with missingness with > 2% heterozygosity > |0.2|, and failing sex checks, or blood relatives were removed; variants with significant deviation from Hardy-Weinberg proportions (p<1x10^-6^ in controls and p<1x10^-10^ in PTSD cases) were also excluded. Imputation to the 1000 Genomes phase 1 reference (19) was performed within the Psychiatric Genetic Consortium (PGC) pipeline (7) using SHAPEIT for phasing (20) and IMPUTE2 for imputation (20, 21). Ancestry principal components (PCs) were calculated according to the PGC guidelines (16).

### Genome-wide association analyses

Genome-wide association analysis (GWAS) for H-SMI was performed using additive logistic regression (PLINK 1.9) and covarying for five ancestry principal components and sex (primary model). A secondary analysis was performed, adding age and employment status as covariates. To address possible bias due to the higher percentage of females, we separately repeated the same analysis as the primary model in females and males (sensitivity analysis). Furthermore, we performed additional GWASs using for the outcome schizophrenia or suicide attempts and the same covariate as the primary model.

QQ plots were used to determine the deviation of the observed p-values from the expected p-values. The genome-wide significance level was set to the standard GWAS threshold of 5×10^-8^. To identify the closest genes associated with each variant, we used the R package *HI Annotator* (22).

Independent loci were first identified based on Locus Zoom (23) observation and Functional Mapping (FUMA) analyses (24) using the African population (AFR) as a reference. Then, we performed logistic regression for H-SMI, selecting the single locus of interest (rs402528) and including as a covariate, beside the first 5 PCs and sex, the second possible independent variant (rs362514). Hillview (25) was also used to identify the linkage disequilibrium (R^2^) between the variants in the HLA locus in our cohort.

### Human leukocyte antigens imputation

HIBAG software package for R was used for the prediction of 4-digit alleles of 7 HLA genes (A, B, C, DQA1, DQB1, DRB1, DPB1) from genotype data (26). HLA imputation was performed as described previously (27). Briefly, we used published parameter estimates for multi-ethnic populations run on the Illumina HumanOmni1-Quad array, which gave the highest Bayesian posterior probabilities after imputation. To reach higher accuracy, we used a call threshold of 0.5 and removed all samples that had imputed alleles with posterior probabilities lower than 0.5, as suggested by Zheng, Shen (26). In other words, if the call threshold was lower than 0.5 for a given sample, we removed the sample from the analysis.

The genotypes of the 87 common HLA alleles were encoded as dichotomous variables for the subsequent analysis under a dominant genetic model, commonly preferred to allelic or recessive models to assess *HLA* associations (28). The association of HLA alleles with lifetime PTSD was evaluated using logistic regression, controlling for the first five principal components for ancestry and sex. A Bonferroni correction was used to adjust for multiple testing in each HLA gene [p<0.0035 for 14 HLA-A alleles (0.05/14), p<0.0031 for 16 HLA-B alleles (0.05/16), p<0.0041 for 12 HLA-C alleles (0.05/12), p<0.005 for 10 HLA-DQA1 alleles (0.05/10), p<0.09 for 11 HLA-DQB1 alleles (0.05/11), p<0.0033 for 15 HLA-DRB1 alleles (0.05/15) and p<0.0056 for 9 HLA-DPB1 alleles].

### Polygenic risk scores

Polygenic Risk Score (PRS) evaluates the genetic liability of several variants into a single per-individual score. To evaluate overlapping genetic risks between H-SMI and other psychiatric disorders prevalent in our cohort (SCZ and MDD) or subcortical brain structures, we used summary statistics from two independent datasets for mental illnesses: the schizophrenia dataset (29), and MDD (MDD2018_ex23andMe) (30); and from seven magnetic resonance imaging-derived brain volumes (amygdala, acumens, brainstem, caudate, pallidum, putamen, thalamus) (31). PTSD-PGC was not analyzed due to its overlap with the GTP-H-SMI dataset.

Risk scores were obtained using the PRSice (v2.0) software (32). SNPs in linkage disequilibrium (*r* ^2^<0.2 within a 500 kb window) were excluded. For each set of SNPs, the PRS score was then calculated as their weighted sum of per loci risk allele in the base dataset: PRs=∑ (Si×Gij)/Mj, where Si the summary statistics for the effective allele for the genotype I, Gij is the genotype I for the j individual (0,1,2), and Mj is the number of alleles included in the PRS of the jet individual. To adjust for variants in linkage disequilibrium with each other, we performed clumping in the discovery population with a window of 500kb and r^2^ of 0.2. To evaluate the association between a set of PRS scores and hospitalization, we used a logistic regression model, adjusting for sex and the first 20 ancestry PCs (the higher number of PCs used in these analyses was to further adjust for stratification to better compensate for the different ancestry between discover and target population). The SCZ-PRS (N=150,064), MDD-PRS (N=173,005), and subcortical brain structure PRS (amygdala N=34,431, accumbent N=32,562, brainstem N=28,809, caudate N=37,741, pallidum, putamen, and thalamus N=34,413) were calculated at a p-value threshold between 0 and 0.5, with a 0.01 increment for a total of 50 different p-values. We considered the two psychiatric disorders (MDD and SCZ) and the seven brain morphologies (amygdala, accumbent, brainstem, caudate, pallidum, putamen, thalamus) as two different separated hypotheses in the PRS analyses; hence, the Bonferroni corrected p-value was adjusted for two psychiatric traits [p=0.0005 for 0.05/(50x2)], and seven brain structures [p=0.0001, for 0.05/(50x7)]. Due to the lack of large summary statistics available for psychiatric diseases in the AA population, PRSs were calculated across ancestry (European for MDD, SCZ, and brain structures -discover population -; AA for the GTP dataset -target population). Hence, to support these results, we re-calculated PRS using PRScsx (33). PRScsx employs a high dimensional Bayesian regression by placing continuous shrinkage (cs) priors on effect sizes of SNPs, enabling multivariate modeling of local linkage disequilibrium (LD) patterns, all of which should improve cross-population accuracy in predicting polygenic risks. As the reference panel should match the GWAS rather than the target population (33), we used 1000 Genomes EUR for LD reference and a phi to 10^-2^ for highly polygenic traits. PLINK 1.9 was used to score the PRS, which was tested for association using logistic regression, including 20 PCs per PRSice. The proportion of phenotypic variance (R^2) accounted by the PRS was calculated on the liability scale (34), assuming a 4.3% prevalence of hospitalization for African Americans (35).

### Gene-based analysis

Gene-based analysis of the GWAS result was also performed using MAGMA (36), as implemented in FUMA (24), as specified above. Input SNPs were mapped to 19,100 protein-coding genes, implemented in FUMA.

### H-SMI genetic correlation and heritability

GCTA (37) was used to estimate the H-SMI heritability by computing the proportion of variance in a phenotype explained by all GWAS SNPs (i.e., the SNP-based heritability) (GREML) (38), adjusting for 10 Ancestry PCs.

Using GCTA, we estimated unbiased genetic correlation between pairs of psychiatric traits in the AA-GTP cohort using a linear mixed model in a series of bivariate analyses (Bivariate GREML) (39)

## Results

### Sociodemographic characteristics

**Table 1** shows the sociodemographic characteristics of the SMI cohort. From the Grady Trauma Project cohort with genome-wide data, we identified 5157 African American individuals, of whom 13.4% (N=690) had psychiatric disease severe enough to require inpatient care (H-SMI). The H-SMI and control groups did not vary by education (p=0.1) or sex (p=0.77). However, the age distribution was different between the two groups (p<0.001; **Supplementary Figure 1**). Similarly, the percentage of unemployment was significantly higher in the SMI group compared to the controls.

**Table 1:** Sociodemographic characteristics of the cohort.

	H-SMI (n=690)	Controls (n=4467)	P-Value
**Age, mean (SD)**	43.07 (11.9)	39.38 (14.1)	<0.001
**Sex (Females)**	75%	75.5%	0.78
**Education**	High School or Below 58.7%	High School or Below 56.4%	0.1
	Some college 33.5%	Some college 33.2%	
	College Graduate 7.8%	College Graduate 10.4%	
**Employment** **Yes**	19%	34%	<0.001

Significance was assessed with fisher’s exact test for binary variables and with the Mann-Whitney U for age.

Multiple correction p-value 0.0125.

The control group of individuals never hospitalized for SMI included some subjects (N=1414) who experienced milder forms of psychiatric illnesses but did not require hospitalization; 3053 individuals did not report any psychiatric illnesses (**Supplementary Table 1**). After accounting for multiple comparison corrections, out of the nine psychiatric disorders, six (suicide attempts, lifetime and current PTSD, lifetime MDD, drug use, and SCZ) were significantly more prevalent in the SMI group compared to the controls (**Supplementary Table 1**).

We observed a high degree of phenotypic correlation among the different psychiatric disorders, except for lifetime alcohol-use disorder; H-SMI showed a similar significant association with all the different psychiatric traits (**Supplementary Figure 2**, **Supplementary Table 2**).

### GWAS of hospitalization due to psychiatric illnesses identified a cluster of risk variants in a chromosome 6 locus near the *HLA* region

H-SMI status was determined as patients who were hospitalized for treatment of various psychiatric disorders (n=690) and were compared with demographically matched controls (n=4467) (**Table 1**). GCTA SNP-heritability of H-SMI () was 14.6% (SE 0.09, p=0.05), comparable to other psychiatric disorders: suicide attempts ~4% (40, 41), depression ~9% (42), neuroticism ~10% (43), bipolar 10% (44), schizophrenia between 15 and 24% (44, 45) and PTSD between 10 to 45% (7, 46, 47). Although limited by the small sample sizes, we observed some degree of genetic correlation between H-SMI and other psychiatric disorders and among some different psychiatric traits (**Supplementary Table 3**).

**Figure 1:**
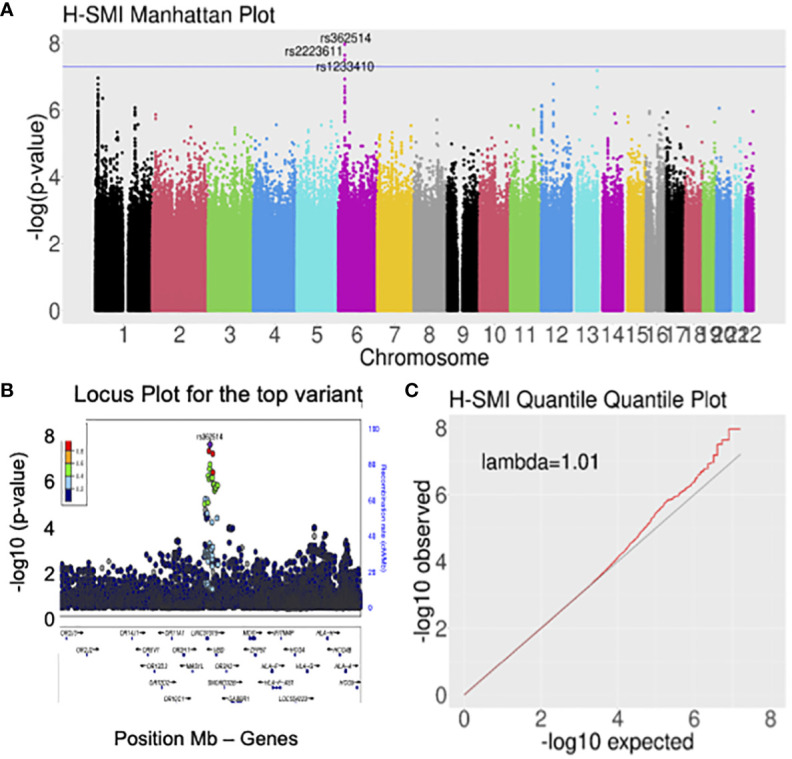
Genome-wide analyses for H-SMI identified a susceptible locus on chromosome 6, within the HLA locus. **(A)** Manhattan plot showing the strength of association between H-SMI and all common variants in the genome, divided by chromosome: a locus on chromosome 6 reached genome-wide significance. Each dot represents a common variant; in the y-axis, the -log_10_ p-value is plotted; the x-axis shows the genomic coordinates of each variant arranged by chromosomes. **(B)** Locus plots showing the variant with the lowest p-value (rs362514): the y-axis shows the –log_10_ p-value; the x-axis is the base pair position (hg19). Circles represent individual SNPs color-coded by LD (r^2^). **(C)** Quantile-quantile (QQ) plot of SMI identified no evident inflation of the p-values. Each dot represents a SNP. On the y-axis a -log_10_(p-value) of the observed data is shown, while in the x-axis is shown the expected -log_10_ (p-value).

Logistic regression of genome-wide data was used to assess the association with H-SMI (**Table 2**, **Supplementary Table 4**). GWAS revealed a genome-wide significant association at the 6p22.1 locus, in the ubiquitin D (*UBD/FAT10*) gene, and around the HLA gene clusters (**Figure 1**) (top variant rs362514, p=1.2x10^-8^, OR=1.4, 95%CI [1.24,1.57]; **Figure 1B**). The quantile-quantile (QQ) plot of the H-SMI-GWAS (**Figure 1C**) showed no evidence of inflation (genomic inflation factor λ=1.01). Given the high percentage of females in this cohort, to evaluate if the significance was driven by them, we repeated the analysis separately by sex. The same locus reached almost genome significance when the same analysis was performed in females only (rs362514, p=6.6x10^-8^ OR=1.4, 95%CI [1.3,1.6]) (**Supplementary Table 4**, **Supplementary Figure 3A**). The same variant (rs362514) reached a nominal significance (p=0.03, OR=1.3, 95%CI [1.01,1.63]) in males (**Supplementary Table 4**), with the same direction of effect in both sexes.

**Table 2:** Top significant variants associated with H-SMI.

SNP	CHR	BP	OR	95%CI	P-Value	Nearest Genes
**rs362514**	**chr6^a^**	**29507310**	**1.40**	**1.25,1.57**	**1.09E-08**	**GPR53P^b^/UBD/FAT10**
rs2223611	chr6^a^	29504206	1.39	1.24,1.55	2.30E-08	GPR53P^b^//UBD/FAT10
rs1233410	chr6^a^	29515494	1.38	1.23,1.55	3.13E-08	OR2I1P
**rs9521945**	**chr13**	**111448374**	**1.51**	**1.30,1.76**	**6.65E-08**	**LINC00567**
**rs6657877**	**chr1**	**12320949**	**1.52**	**1.30,1.78**	**1.11E-07**	**VPS13D**
rs446198	chr6^a^	29507426	1.40	1.23,1.58	1.20E-07	GPR53P^b^//UBD/FAT10
**rs73305449**	**chr12**	**54136144**	**1.70**	**1.39,2.08**	**1.69E-07**	**RP11-686F15.3**
rs60771497	chr1	12320273	1.51	1.29,1.77	1.70E-07	VPS13D
rs419957	chr6^a^	29505769	1.39	1.23,1.57	1.99E-07	GPR53P^b^//UBD/FAT10
rs9521944	chr13	111448347	1.48	1.28,1.72	2.09E-07	LINC00567
rs72864994	chr1	12329511	1.50	1.29,1.75	2.48E-07	VPS13D
rs387659	chr6^a^	29516180	1.35	1.20,1.51	2.56E-07	OR2I1P
rs426595	chr6^a^	29512482	1.38	1.22,1.56	3.09E-07	GPR53P^b^//UBD/FAT10
rs17037973	chr1	12359872	1.57	1.32,1.87	3.30E-07	VPS13D
rs7546794	chr1	12336147	1.59	1.33,1.89	3.85E-07	VPS13D
rs2745408	chr6^a^	29501920	1.38	1.22,1.56	4.36E-07	LINC01015
rs144275028	chr1	12370292	1.51	1.28,1.76	4.48E-07	VPS13D
**rs183313357**	**chr1**	**33012056**	**2.08**	**1.56,2.76**	**4.52E-07**	**ZBTB8A**
rs73305452	chr12	54137477	1.67	1.37,2.04	5.20E-07	RP11-686F15.3
rs398616	chr6^a^	29514230	1.37	1.21,1.55	5.52E-07	OR2I1P

**SNP,** Single Nucleotide Polymorphism; **CHR,** Chromosome; **BP,** base pair position (hg19); **OR**, odd ratio; **95%CI,** 95% confidence intervals; **P-Value,** association p-value**; Nearest Genes,** Nearest genes defined by using hiAnnotator (22).

^a^ Near HLA Locus; ^b^Pseudogene.

In bold, independent SNP variants at clump-r2 threshold of 0.5 and physical distance threshold 250Kb (PLINK 1.9). A more extensive table with all nominal significant variants (p<10^-6^) is shown in **Supplementary Table 4**.

In males, we observed a few variants with genome-wide significant association, but given the limited number of individuals (173 cases and 1096 controls), we could not infer any conclusion (**Supplementary Table 4**, **Supplementary Figure 3B**).

A secondary analysis adjusting for age and employment did not change the results, identifying an additional locus on chromosome 13 (**Supplementary Figures 4A, B**). GWAS of SCZ (237 cases and 4891 controls) and suicide attempts (669 cases and 4475 controls) were also reported (**Supplementary Figures 4C, D**) for comparison with the GWAS of H-SMI (cases and controls reported here are different from **Supplementary Table 1** because they refer to the entire AA GTP cohort, including samples with missing H-SMI data).

The association in the 6p22.1 locus seemed to involve two independent signals, rs362514, the top variant in the primary analysis (**Figure 1C**), and rs402528 (p=1.6x10^-6^, OR=1.4, 95%CI [1.20,1.54] (**Supplementary Figure 5**, **Supplementary Table 4**), possibly implicating different genes in the same locus (linkage disequilibrium R^2^ between the two variants = 0.56; **Supplementary Figure 5B**).

However, to verify that we indeed observed two different signals, we repeated the analyses for rs402528, covarying for the main variant (rs362514): we did not observe any significant association (p=0.5, OR=1.0, 95%CI [0.88,1.29]) indicating that we are probably in the presence of a single signal.

We evaluated whether either of these two variants was significant in other GWAS of psychiatric disorders, and most specifically in GWAS for bipolar, MDD, SCZ, and suicidality (**Supplementary Table 5**); unfortunately, neither of these 2 SNPs were present in the summary statistics of the cross-disorder group (48). rs362514 was found to be of nominal significance in the GWAS meta-analysis for BD of 32 cohorts from Europe, North America and Australia (p=0.023 OR= 0.97) (49) and in the GWAS combined meta-analysis for major depressive disorder of PGC29 with five expanded samples (decode, Generation Scotland, GERA, psych, and UK Biobank, excluding 23 and me) (p=0.042 OR=0.98) (30). Both rs362514 and rs402528 were found to have nominal significance in the GWAS for bipolar (N=20,129) or bipolar and SCZ (N=33,426) vs. controls (N=54,065) (50), in the GWAS of SCZ in the European cohort (33,640 cases and 43,456 controls) (45) and in the GWAS of the Depression and Worry Cluster (43). However, these two variants did not show any association in the main GWAS for suicide attempts (41), neuroticism, or depressed affect subclustered phenotypes (43).

It is interesting to note that these SNPs seem to increase the risk of H-SMI in the current study (OR>1); however, in the other reported external studies, they seem to reduce the risk of the disorder/trait (OR<1).

To evaluate the direct association between these two genetic variants and gene expression levels in different tissues, we interrogated the database GTEx (https://gtexportal.org). GTEx data is a comprehensive public resource to study the relationship between genetic variants and gene expression in multiple human tissues and across individuals (84.6% White, 12.9 African American, and 2.5% Others). Based on the GTEx, there is no evidence that rs362154 or rs402528 are eQTL for the *UBD* gene. However, they seem to regulate expression levels of different HLA genes (e.g., eat for HLA-H, HLA-J, HLA-A. HLA-G) in multiple tissues (**Supplementary Table 6**).

Notably, gene expression was evaluated in a dataset including a high percentage of White individuals, while our study focuses on AA samples.

In the primary analysis, the association test statistics for the GWAS revealed a few other exciting loci in chromosomes 1, 12, and 13 (**Figure 1A**), but none of those reached genome-wide significance. After additional adjusting for age and employment status (secondary analyses), few SNPs on chromosome 13 reached genome-wide significance, with the leading SNP with the lowest p-value (rs9521945) being located in an intergenic region within *ING1* and *LINC00346* & *ANKRD10* (**Supplementary Figure 4B**).

### The gene-based analysis revealed additional loci associated with H-SMI

**Figure 2:**
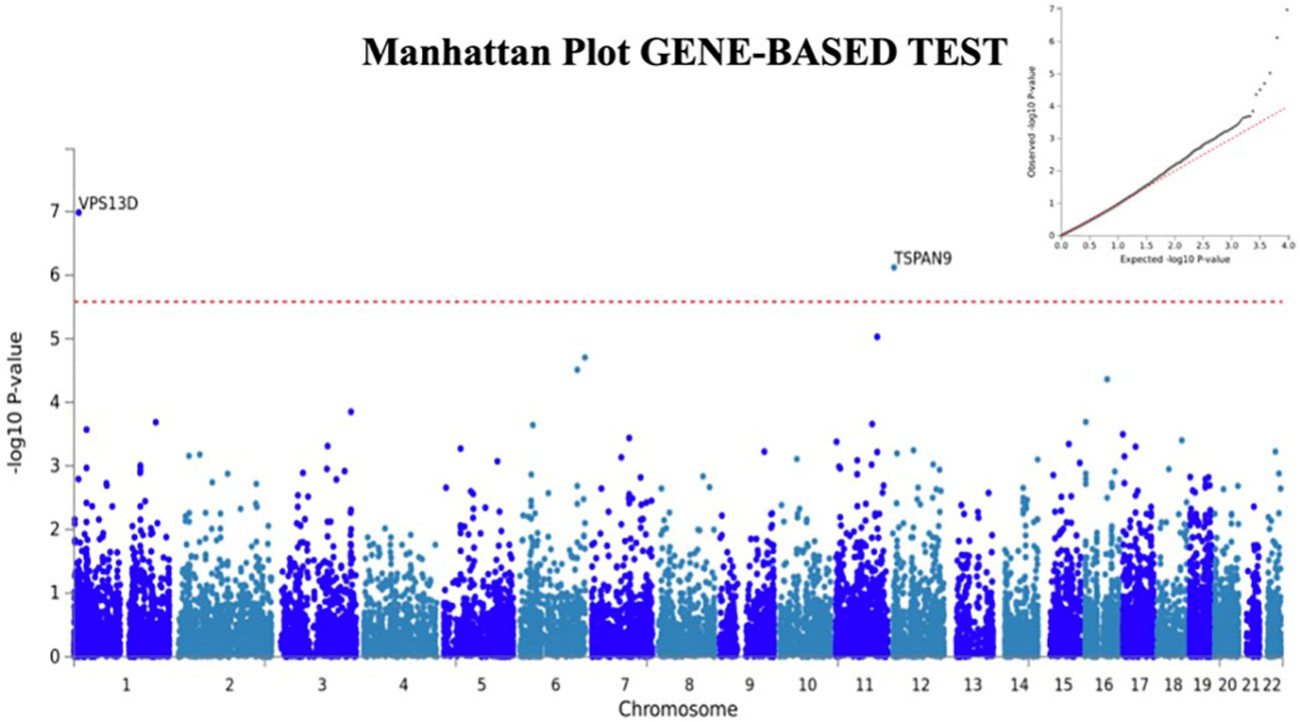
Manhattan plot for Gene-Based analyses generated by FUMA. Two additional genes, *TSPAN9* and *VSP13D* are associated with H-SMI phenotype at a genome-wide significance. Input SNPs were mapped to 19100 protein-coding genes. Genome-wide significance (red dashed line in the plot) was defined at P = 0.05/19100 = 2.618x10^-6^. In the right corner, the QQ plot for the gene-based test is shown.

We next used FUMA (24), a tool to functionally map and annotate results from GWAS, to perform additional analyses using the H-SMI summary statistics. Gene-based analyses (MAGMA) revealed two additional genes, *VSP13D* (chromosome 1; p=3.6x10^-8^) and *TSPAN9* (chromosome 12, 4.3x10^-7^) associated with H-SMI at a genome-wide significance level (**Figure 2**).

### *HLA* locus is associated with severe mental illness for hospitalization

To further explore the association between the H-SMI and the HLA locus, we imputed HLA alleles for the GTP population (**Supplementary Table 7**) and evaluated their association with H-SMI. Among the 7 HLA genes tested, three alleles reached nominal significance, two of which HLA-A*23:01 (OR=1.04, p=2.3x10^-3^) and HLA-C*06:02 (OR=1.04, p=1.5x10^-3^) survived Bonferroni correction when adjusting for multiple testing in each HLA gene (p<0.0036 for 0.05/14 and p<0.0042 for 0.05/12, respectively).

### Lack of association across ancestry between psychiatric disorders and subcortical brain structure polygenic risk scoring and H-SMI

To evaluate common risk variants between H-SMI and other psychiatric diseases, we use the PRS from PGC-SZ (29) and depression (30). When using polygenic scoring with a traditional method (PRSice), we observed nominal significance at several p-value threshold levels (**Supplementary Figure 6**); the lowest p-value for SCZ and MDD was, respectively, p=0.018 (R^2 =^ 0.20%) and 0.013 (R^2 =^ 0.22%). **Supplementary Table 8** includes information about the initial number of SNPs from summary statistics, after clumping, and the one associated with the best p-value. Given the difference in ancestry between the target dataset (AA GTP-H-SMI) and the training datasets (European PGC-SCZ and MDD), we decided to confirm the results using a different method (PRScsx), which has been designed to be less biased across ancestry: no significant association was found using this method (**Supplementary Table 9**).

Interestingly, when we performed both analyses with subcortical brain structures, we observed a nominal significant association between amygdala and H-SMI with both methods (**Supplementary Figure 7**, **Supplementary Tables 8**, **9**); no other brain structures were associated with H-SMI.

## Discussion

In this study, we identified a susceptible locus on chromosome 6, near the HLA genes, which increased the risk of hospitalization in individuals with severe psychiatric disorders. Severe mental illnesses (SMI) represent a group of psychiatric disorders in which the symptoms are often so significant that the patients may require long-term care and hospitalization. Being able to identify specific mechanisms that are involved in SMI may facilitate the identification of at-risk patients for early intervention, decreasing both individual suffering and overall public health impact. Furthermore, the shared genetic heritability across many psychiatric disorders suggests that examining transdiagnostic severity of SMI may be a powerful approach. To our knowledge, no genetic analyses specifically on SMI have been performed to date.

The subjects for this study were part of a larger investigation of genetic and environmental factors that predict the response to stressful life events in a predominantly African American urban population of low socioeconomic status (13): the Grady Trauma Project or GTP. The goals of this specific study were first to verify the heritability of hospitalization caused by severe mental illnesses (H-SMI) and then to identify possible novel genetic associations related to H-SMI. Indeed, the SNP-heritability of H-SMI was comparable to that observed in other psychiatric disorders (e.g., PTSD from 10 to 45%) (7, 46, 47).

In our cohort, we also observed a high phenotypical correlation among most of the different psychiatric disorders but not all (e.g., SCID alcohol), most likely due to the small sample representation of those individuals in our dataset, and probably indicating that this trait (alcohol) should not be included in the H-SMI group. We detected some degree of genetic correlation between H-SMI and other psychiatric traits. These results should be considered with caution, given the small sample size. However, several other studies reported a high degree of genetic correlation among many psychiatric disorders (4, 48, 51, 52), noticing that “current clinical boundaries do not reflect distinct underlying pathogenic processes, at least on the genetic level” (51).

GWAS for H-SMI in the GTP sample revealed a significant association in the 6p22.1 locus in the ubiquitin D (*UBD/FAT10*) gene and adjacent to the HLA gene cluster, a complex polymorphic system involved predominantly in regulating immune and inflammatory processes, but also in neuroplasticity by microglia synaptic pruning (53).

When we performed separated GWASs in the same dataset considering as outcomes individuals diagnosed with SCZ (**Supplementary Figure 4C**), suicide attempts (**Supplementary Figure 4D**), or PTSD subjects (16), variants in this locus (6p22.1) did not reach a GWAS-significant p-value, implying that the observed association was not driven by any specific psychiatric disorders but by their cumulative effects.

Two groups of variants, led by rs362514 and rs402528, seemed responsible for the observed H-SMI GWAS significant association in the 6p22.1 locus. Sex-specific analyses lead to comparable results, although not reaching genome-wide significance, indicating that this locus may similarly affect H-SMI in both sexes. The main variant (rs362514), together with another variant in the same loci (rs402528), were also associated with changing expression levels of HLA genes in different tissues (GTEx), indicating that these variants could alter biological processing, causing pathological phenotypes. Despite their physical association, there is no evidence that these variants also regulate the expression of the *UBD* gene. Moreover, these two variants showed nominal significance in several other GWAS of psychiatric diseases, including SCZ, BD, and MDD (30, 43, 45, 49, 50), although in the opposite directions. These variants were also associated with the endophenotype “worry” but not with neuroticism or the depressed affect sub-cluster (43). In that study, both neuroticism and “depressed affect” may be related to a “less severe” cluster of psychiatric symptoms since they appeared significantly correlated with personality and other psychiatric traits (e.g., subjective well-being, ADHD, smoking behaviors), anthropometric traits (e.g., waist-to-hip ratio) or physical health-related outcomes (e.g., longevity, number of children) (43). On the other hand, *worry* was found more significantly correlated to anorexia, SCZ, and BD (43).

All the above studies were performed mainly in populations of European ancestry, while ours focused on an African American population. As noted earlier, the risk allele in the previous analyses seems different from the AA-GTP cohort. This is a well-known phenomenon known as the “flip-flop phenomenon” (54, 55). This phenomenon may occur when the casual variant is located at another locus through interactive effect or linkage disequilibrium. Given the highly polymorphic nature of the HLA gene region, including the complement 4 (56), the different risk allele in the different studies (European vs. AA) may be due to “population variation in interlocks correlation” (54). We cannot also exclude that the different ancestry genetic backgrounds or environments may have caused the heterogeneous effects of the same variants.

Furthermore, the observed nominal significance at this locus for several GWAS from psychiatric disorders might imply that those larger studies were able to capture some of the variance caused by severe symptoms. Indeed, a few other studies have implicated the HLA locus in the more severe spectrum of psychiatric disorders (56–58).

Plausible causative pathways contributing to the worsening of psychiatric symptoms involve dysregulated immune responses instigated or modulated by underlying genetic vulnerabilities (59–61). The role of the immune system in MDD, PTSD, BD, and SCZ is supported by a plethora of epidemiological and laboratory studies (62–72), with some of the first evidence being presented in the early 70s (73, 74). The crucial contributions of HLA and immunogenetics to SCZ and BD have been recently reviewed (58, 75, 76). Additionally, one of the strongest GWAS signals consistently implicated in SCZ resides on chromosome 6p21.32-p22.1, which includes the HLA region (56, 77–80).

The HLA locus has also been associated with the modulation of treatment response in psychiatric patients (81, 82). Some common variants in this locus might contribute to the response to antipsychotic drugs in schizophrenia patients with persistent psychosis (83). These investigations suggest that hospitalized patient samples (including those in our H-SMI group) are enriched with treatment-resistant individuals and genetic pool-resistant variants (e.g., genes in the HLA locus). Future research should aim to empirically test this hypothesis by evaluating the genetic correlation between treatment-resistant patients (e.g., SCZ and MDD) and H-SMI cases.

A few traditional GWAS studies have investigated the role of the functional HLA alleles in psychiatric disease (27, 76). In our study, we specifically imputed HLA alleles using GWAS data and found that H-SMI was nominally associated with two HLA variants, HLA-A*23:01 and HLA-C*06:02, supporting the hypothesis of the plausible interaction between the immune system and severity of mental diseases.

To evaluate common risk variants between H-SMI and other psychiatric diseases, we applied PRS (using two different methods) from PGC-SCZ (29), MDD (MDD2018_ex23andMe), and subcortical brain structures (31). The inconsistency of these results may be caused by the different ancestry populations (discovery and target), making it difficult to draw definite conclusions. However, interestingly, we observed a nominally significant association between PRS of amygdala volume and H-SMI in both analyses (PRSice and PRScsx), possibly implicating this brain structure in the extreme phenotypes associated with hospitalization for mental illnesses.

Finally, gene-based analyses revealed two other genes, *TSPAN9* and *VSP13D*, associated with H-SMI. Note that *VSP13D* was among the top genes in the primary GWAS analyses (**Table 2**). *TSPAN9* is a gene that is remarkably highly conserved during evolution. It is a member of the transmembrane 4 superfamily, whose functions are involved in a wide variety of cellular processes, including the support of the nervous system, the regulation of monocyte fusion, and T-cell proliferation (84). Although the specific functions of *TSPAN9* are still understudied, it is known that this gene a) is expressed in megakaryocytes, which are regulated by cytokines, and b) is a component of the tetracaine microdomains on the ‘platelets’ surface (85), and c) plays a fine-tuning role in platelet activation (86).

Interestingly, besides playing an essential role in preventing excessive blood loss, platelets also have a critical role in inflammation by recruiting leukocytes (85). *VSP13D* is also a highly evolutionarily conserved gene. Mutations in this gene, which encodes chorein, have been associated with recessive ataxia (87), while its genetic variants appeared to regulate IL-6 expression levels and production (88). Thus, VPS13D could be involved in cytokine trafficking and immune response. Hence, gene-based analyses additionally point to the role of an immune response in H-SMI phenotypes.

The major limitation of this study is the relatively modest sample size of the GWAS compared to most modern studies, which now include between 20 thousand and over 100 thousand subjects, depending on the specific traits/disorders. This limited sample size may have obscured other loci involved in H-SMI. Second, the primary analyses were performed in an AA population. Although we reported some evidence that these findings could be extended to other ancestries (e.g., European ancestry), we should use these results with caution and perform additional evaluations before generalizing these data to other populations. Furthermore, we could not use a comprehensive multi-ancestry reference dataset when inferring HLA haplotypes, implying that this method may not perform as well in an African American population and that these results should be used with discretion.

We were also limited in the PRS analyses by the small number of large psychiatric studies performed in AA populations, and the few available contain our data as part of the consortium (7, 16, 34). PRS is sensitive to ancestry and inflation due to overlapping samples between discovery and target population. With new statistical methods becoming available (89, 90), and more studies focused on AA cohorts, we should be able to overcome this limitation in the future. With 8.0% of our H-SMI cases having current PTSD, a PRS study focused on this subgroup is also a future goal as more discovery datasets become available.

We should also consider that some of the psychiatric disorders were self-reported (e.g., schizophrenia) – this is particularly important because African American patients are often misdiagnosed/over-diagnosed with schizophrenia over other underlying causes of distress (91–94). On the other hand, our case cohort, in which “hospitalization for psychiatric disorders” is the phenotype, indicates the presence of a severe mental illness, despite the specific diagnosis. Moreover, the GTP cohort is medically underserved and less likely to have access to psychiatric diagnosis. Importantly, H-SMI is not a clinical diagnosis but an empirical phenotype, possibly not generalizable to other cohorts. These limitations could be viewed from a different perspective: considering both misdiagnosis and lack of psychiatric evaluations, the empirical SMI (and hospitalization) allows us to focus on severe disorders in this cohort while recognizing that lack of diagnosis is not equivalent to lack of disorders; and enables us to combine different disorders, independently from their specific diagnosis (or misdiagnosis).

## Conclusion

It is becoming clear that patients with different psychiatric diseases have overlapping genetic risks that cannot be revealed by categorizing them using classical nosological definitions. In this study, we empirically identified a group of patients for whom the severity of these diseases (e.g., SCZ, BD, and MDD) may have amplified a signal at the HLA locus, implicating the immune system and the immune response in the development of severe transdiagnostic H-SMI. Clustering patients based on their genetic risks might help to identify novel transdiagnostic approaches to severe mental illness.

## Data availability statement

The original contributions presented in the study are publicly available. The genotype data can be found here: https://www.ncbi.nlm.nih.gov/projects/gap/cgi-bin/study.cgi?study_id=phs002046.v1.p1.

## Ethics statement

The studies involving humans were approved by Emory University School of Medicine and Grady Memorial Hospital, Atlanta, Georgia. The studies were conducted in accordance with the local legislation and institutional requirements. The participants provided their written informed consent to participate in this study.

## Author contributions

AL designed and performed the main analyses and wrote the draft of the manuscript. BP and KR helped with data analysis and interpretation, drafting and performing a critical revision of the manuscript. SK performed the HLA analyses and helped revising the manuscript. SC, RH, CG, BB performed data collection and diagnosis, phenotype cleaning and helped with the revision of the manuscript. AW, TJ, VM, ED, and AS helped with data analyses, writing, and revising the manuscript. All authors contributed to the article and approved the submitted version.
