# Supplementary material for: Genetic risk for hospitalization of African American patients with severe mental illness reveals HLA loci
Source: Front Psychiatry. 2024 Feb 26;15:1140376. doi: 10.3389/fpsyt.2024.1140376 (PMC10925622; doi:10.3389/fpsyt.2024.1140376)
Supplement: Supplementary file 1 [file DataSheet_1.zip › SupplementalFigures_F_20204.docx]

**Supplementary Figures**

**Supplementary Figure 1. Different age distribution in HSMI vs. Controls.**

**Controls**

**H-SMI**

**Supplementary Figure 1. Age distribution in H-SMI and controls**

**­­**

**Supplementary Figure 2. Correlation plot between H-SMI and other psychiatric disorders.**
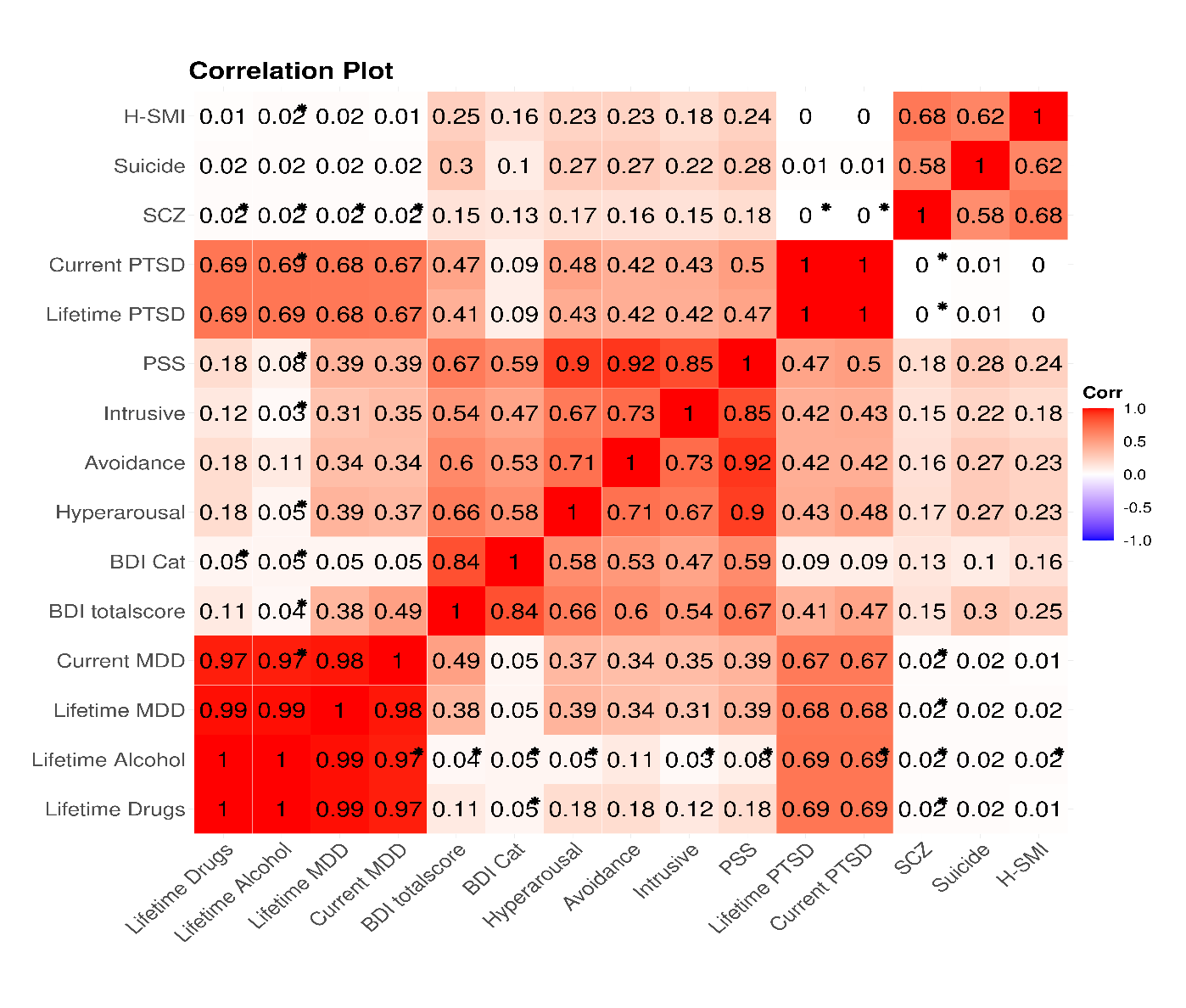


**Supplementary Figure 2.** Correlation Plots showing high levels of correlation within the different psychiatric illnesses, including H-SMI. The color denotes the magnitude and direction of the correlation. The association values are also reported inside each square. **Stars denote not significant p-values**. Some high association values (e.g., 0.9) did not reach the p-value of threshold significance (see Supplementary Table 2). Among nominal variables association was calculated using Cramer’s V and p-values using X-square; between numeric variables a Spearman correlation was used, and between nominal and numeric an Anova analysis was used. The color denotes the magnitude and direction of correlation. The association values (numbers inside each square) are also reported.

.

**
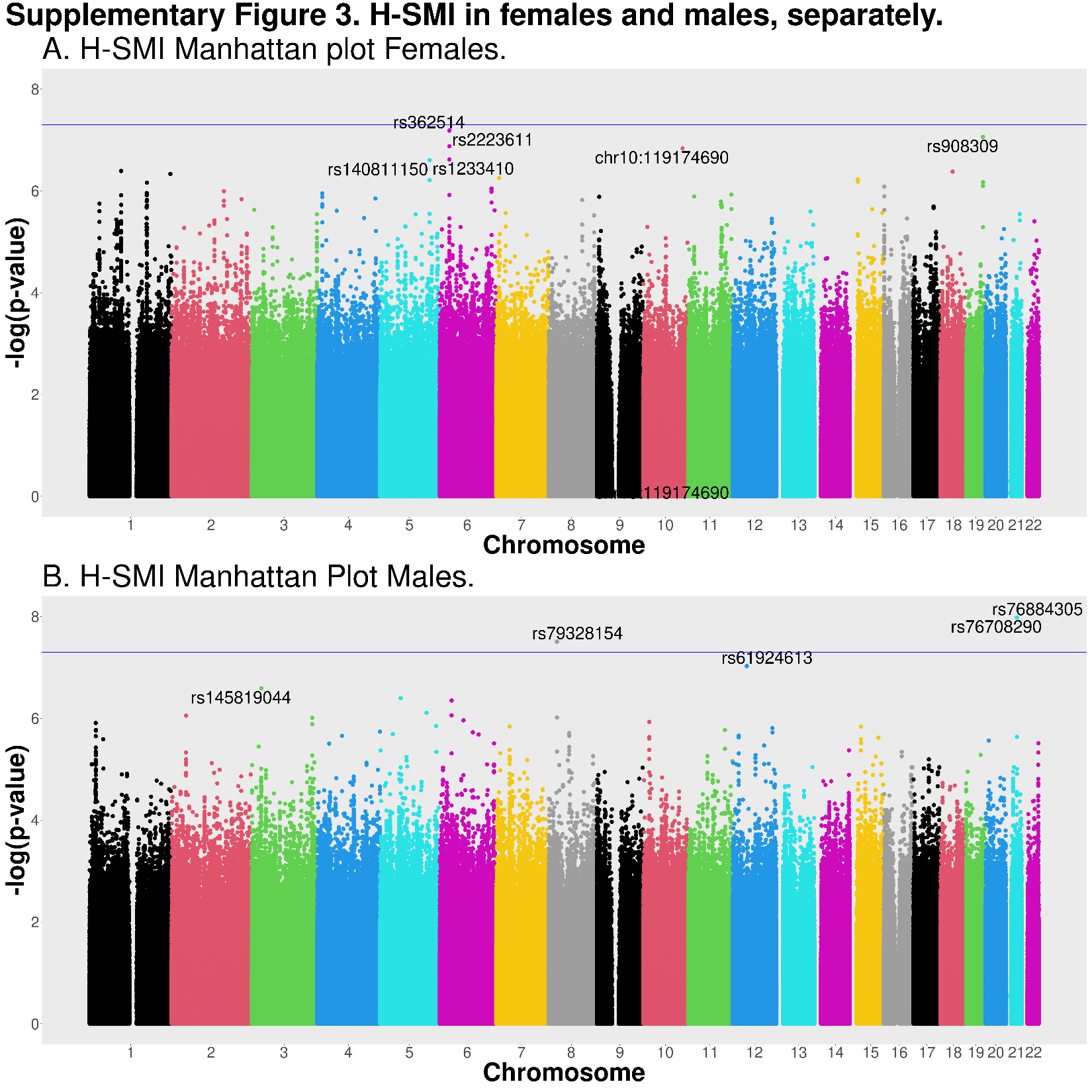
Supplementary Figure 3.** Manhattan plot showing the association of H-SMI in Females (A) and Males (B) separately. Each dot represents a common variant; in the y-axis, the -log_10_ p-value is plotted; the x-axis shows each chromosome Although we observed several variants with genome-wide significance in males, the sample size is too small to draw any conclusion. Suggestive significant variants were reported (Supplementary Table 4) for future reference.

**Supplementary Figure 4. Exploring the GWAS of SMI.**

**A. H-SMI adjusted for age, sex, employment and 5 PCs.**

**B. Chromosome 13 Locus Plot.**


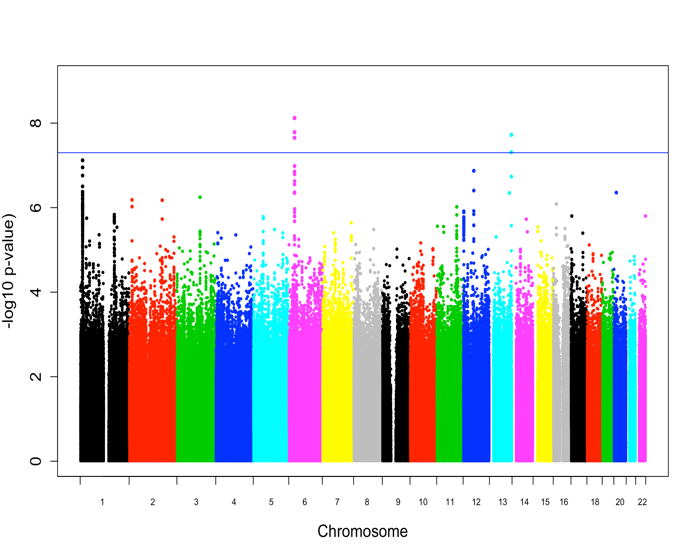


**D. Suicidal Attempts.**

**D. Suicidal Attempts**

**C. Schizophrenia Treatments.**


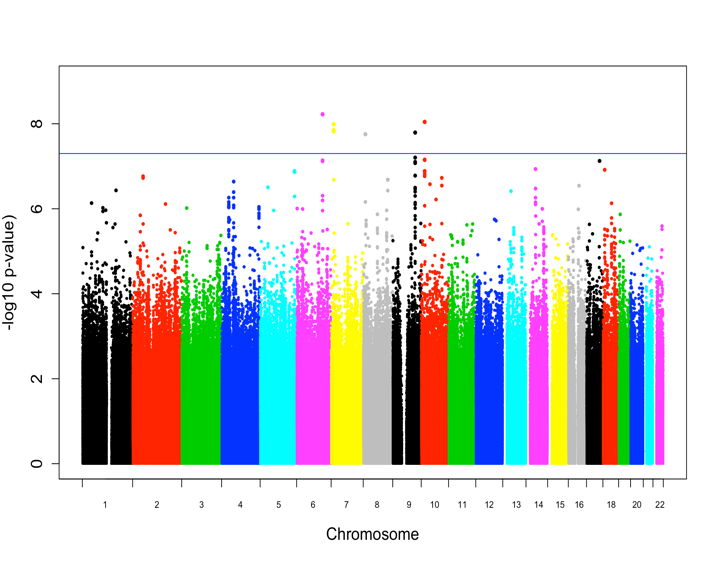

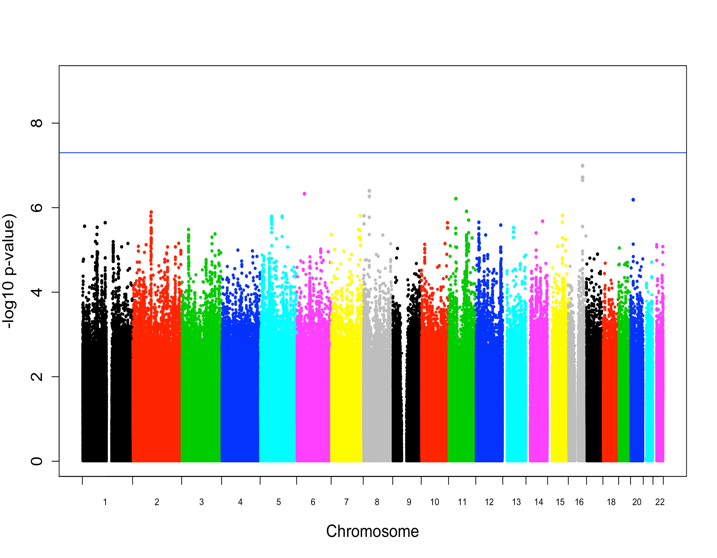


**Supplementary Figure 4.** Manhattan plot showing association of common variants in the genome with a) SMI adjusted for 5 PCs, sex, age, and employment and b) its relative Chromosome 13 Locus Plot c) Manhattan plot of schizophrenia treatment; and d) suicidal attempts. Each dot represents a common variant; in the y-axis, the -log_10_ p-value is plotted; the x-axis shows each chromosome.

**Supplementary Figure 5. Locus plots identified a possible independent variant associated with H-SMI.**

1. **Locus Zoom.**

**B. Linkage Disequilibrium Plot of the suggestive significant variants in the HLA locus.**

**
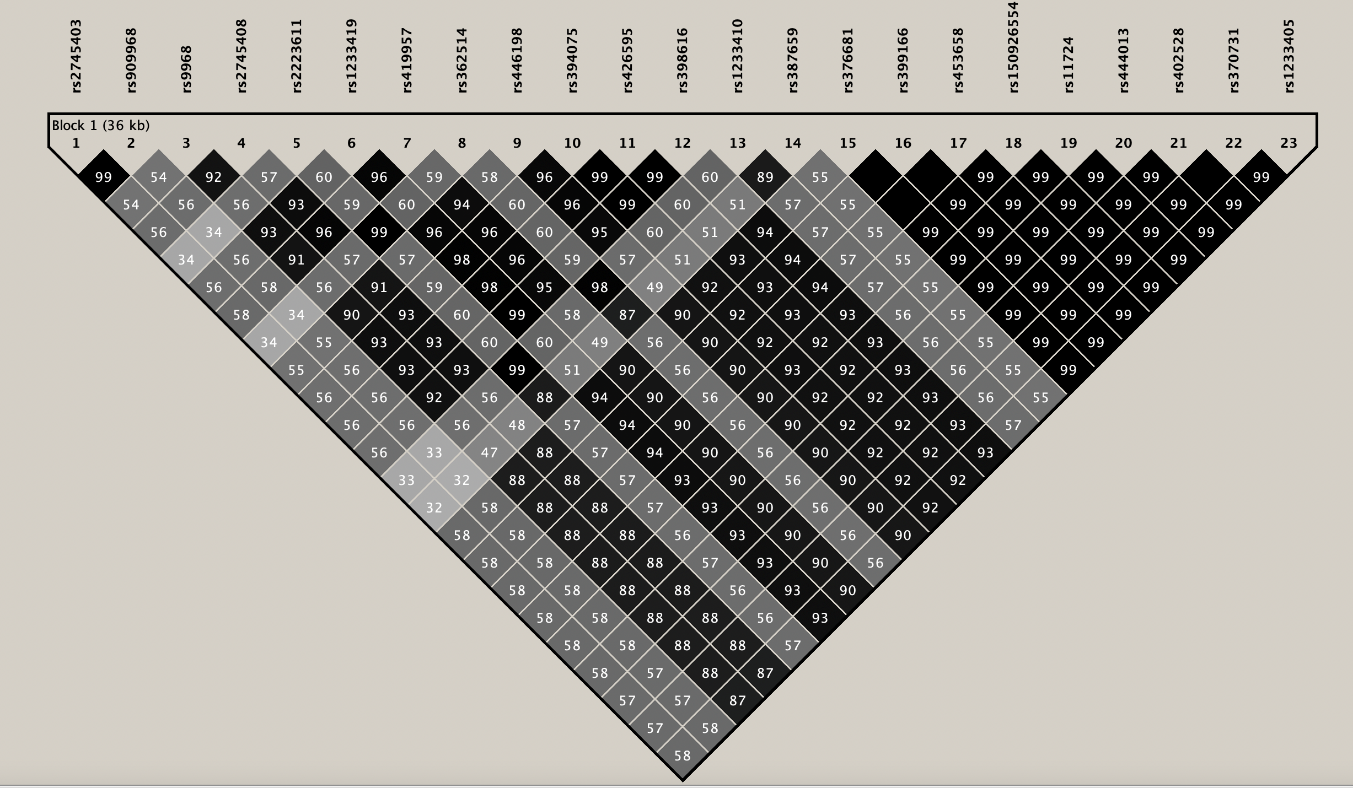
**

**Supplementary Figure 5.** **A. Locus plot:** y-axis shows the –log_10_  p-value; x-axis is the base pair position (hg19). Circles represent individual SNPs color-coded by LD (r^2^). While figure 1B of the main text shows the LD for the leading SNP with the lowest p-value (rs362514), this locus plot shows a second variant, rs402528 (p=1.3x10^-6^), in the ubiquitin D (*UBD/FAT10*) gene, clustering together with a second group of SNPs, all in strong LD within each other. **B. Haploview**. Linkage Disequilibrium plot showing the 23 variants (Supplementary Table 4) in the HLA locus associated with H-SMI. Rs362514 and rs402528 are circled*.* The degree of association is shown based on the gray color. Inside each square the LD-R^2^ between two variants is shown. Rs362514 and rs402528 have a R^2^=0.56.

**Supplementary Figure 6. Polygenic Risk Score of Mental Disorders and H-SMI Across Ancestry.**


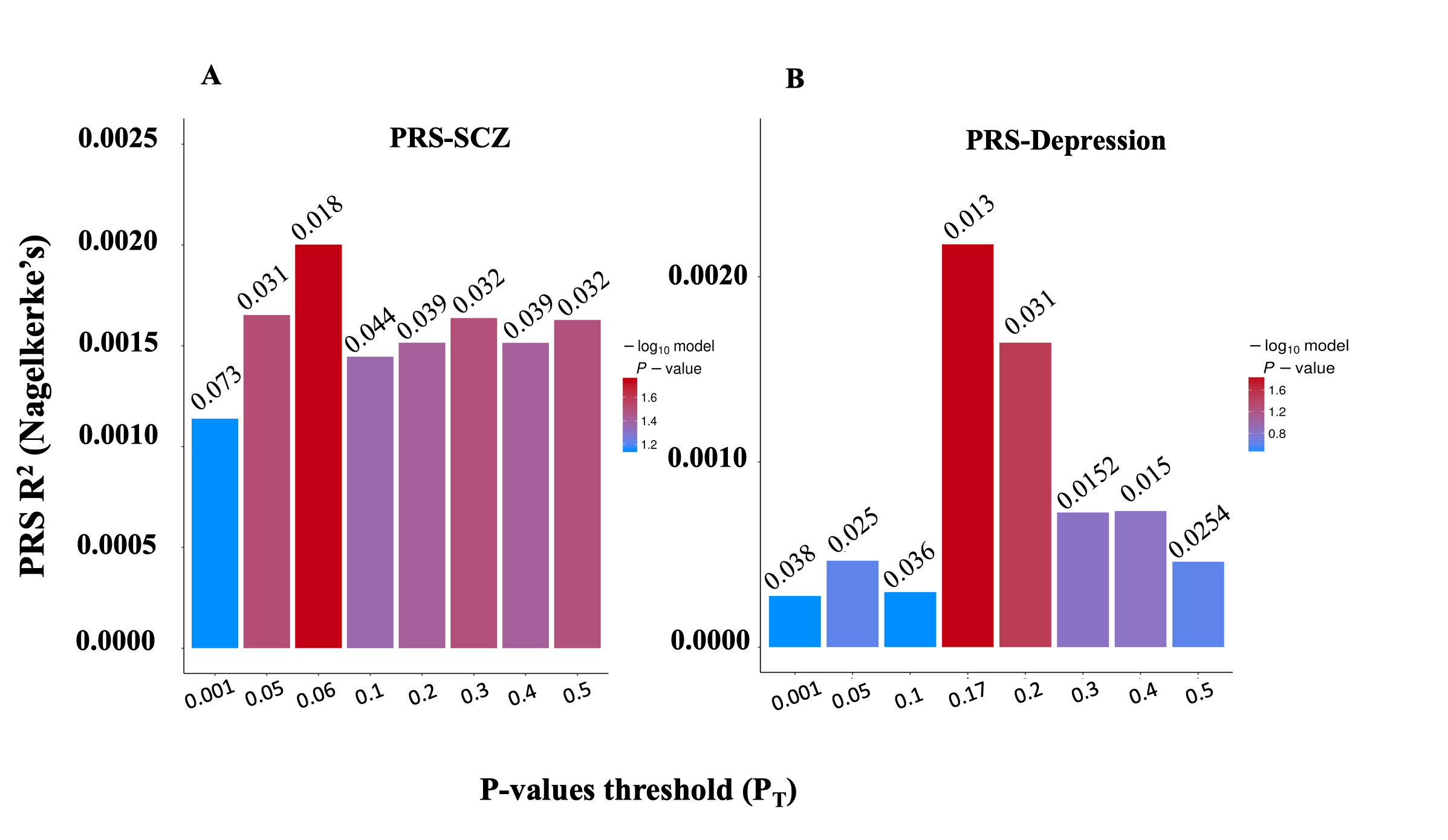


**Supplementary Figure 6. Polygenic Risk Score of Mental Disorders and H-SMI Across Ancestry.**

Bar levels displaying the model fit (R^2^), for each corresponding p-values threshold (PT). Bar plots from PRSice showing results at broad P-value bins (P_T_) for schizophrenia (A) and depression (B) from the PGC predicting H-SMI at nominal levels. On the x-axis, eight representative p-value bins (out of 50) are shown, including the one with the lowest p-value (P_T_=0.06 for A, and P_T_=0.17 for B). The y-axis shows the portion of the variance explained for each bar (Nagelkerke R^2^). The bars are color-coded by -log_10_ p-value.

**Supplementary Figure 7. Polygenic risk scores of subcortical brain structures may reveal an association between H-SMI and amygdala.**

**
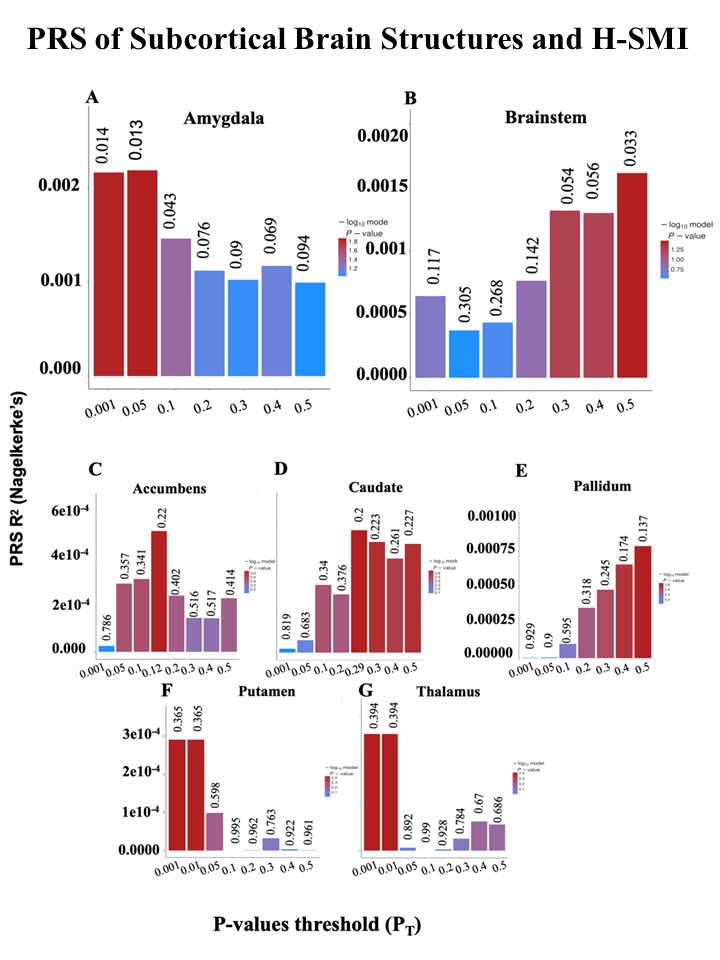
**

**Supplementary Figure 7. H-SMI shares polygenetic risks with Amygdala.** Bar levels displaying the model fit (R^2^), for each corresponding p-values threshold (PT). Bar plots from PRSice showing results at broad P-value bins (P_T_) for amygdala (A), brainstem (B), accumbens (C), caudate (D), palladium (E), putamen (F), and thalamus (G) from the Enigma summary statistic data. On the x-axis, eight representative p-value bins (out of 50) are shown, including the one with the lowest p-value association (P_T_=0.06 for A, and P_T_=0.17 for B). The y-axis shows the portion of the variance explained for each bar (Nagelkerke R^2^). The bars are color-coded by -log_10_ p-value.
